# Age-related decline of the acute local inflammation response: a mitigating role for the adenosine A_2A_ receptor

**DOI:** 10.18632/aging.101303

**Published:** 2017-10-18

**Authors:** Cynthia Laflamme, Geneviève Bertheau Mailhot, Marc Pouliot

**Affiliations:** ^1^ Faculty of Medicine, and Centre de Recherche du CHU de Québec - Université Laval, Quebec City, Canada

**Keywords:** aging, neutrophils, migration, apoptosis, adenosine, cytokines

## Abstract

Aging is accompanied by an increase in markers of innate immunity. How aging affects neutrophil functions remains of debate. The adenosine A_2A_ receptor (A_2A_R), essential to the resolution of inflammation, modulates neutrophil functions. We sought to determine whether or not A_2A_R protects against the effects of aging. We monitored neutrophil influx, viability, and activation as well as cytokine accumulation in wild-type (WT) and A_2A_R-knockout mice (KO) at three different ages.

Several readouts decreased with aging: neutrophil counts in dorsal air pouches (by up to 55%), neutrophil viability (by up to 56%), elastase and total protein in exudates (by up to 80%), and local levels of cytokines (by up to 90%). Each of these parameters was significantly more affected in A_2A_R-KO mice. CXCL1-3 levels were largely unaffected. The effects of aging were not observed systemically. Preventing neutrophil influx into the air pouch caused a comparable cytokine pattern in young WT mice. Gene expression (mRNA) in leukocytes was affected, with CXCL1 and CCL4 increasing and with TNF and IL-1∝ decreasing.

Conclusion: Aging has deleterious effects on the acute inflammatory response and neutrophil-related activities, and defective migration appears as an important factor. A functional A_2A_R signaling pathway delays some of these.

## INTRODUCTION

Aging is often associated with a chronic, low-grade inflammation characterized by increases in circulating pro-inflammatory cytokines. Originally coined “inflammaging” by Franceschi et al. [1], this pheno-menon has been linked to age-related disorders and earlier mortality [2, 3]. Aging is also associated with immunosenescence, a progressive deterioration of the adaptive immune system, characterized by reduced responsiveness to preventive vaccination or by increas-ed susceptibility to cancer, autoimmune and infectious diseases. Infections and sepsis rank among the top causes of mortality in the elderly [4].

An early feature of the acute innate response is the recruitment of polymorphonuclear leukocytes (neutro-phils) [5]. In response to specific activating signals, these short-lived cells migrate across the endothelium and often accumulate in large numbers at the site of a lesion, where they contribute to host defenses by phagocytosing pathogens and cell debris, generating cytotoxic oxygen-derived reactive agents, and by releasing proteolytic enzymes and antimicrobial proteins. Inflammatory neutrophils also produce and release an array of specific soluble mediators of inflammation. Eicosanoids, cytokines, and chemokines [6-8] may each influence the course of immune reactions in a multi-pronged fashion, by soliciting and regulating different cell types involved in the normal development and resolution of an effective inflam-matory response [9]. Aging reportedly affects important receptor-driven functions of neutrophils [10]. Extracellular trap formation, phagocytosis, degranula-tion, ROS production and the microbicidal capability of neutrophils all decline with age [11]. However, the effects of aging on these and other innate immune responses remain incompletely understood, particularly regarding the recruitment of neutrophils and their role in the local build-up of cytokines.

Adenosine is an autacoid with a broad spectrum of activities. Its formation increases under conditions such as sepsis, inflammation and hypoxia and plays an important role in the resolution of inflammation [12, 13]. The adenosine A_2A_ receptor (A_2A_R) constitutes a non-redundant physiological negative feedback mechanism that terminates inflammatory responses [14]. Engagement of A_2A_R in young mice inhibits phagocytosis, neutrophil adhesion to endothelial cells and generation of cytotoxic oxygen metabolites. Neutrophils have been identified as an important target of the anti-inflammatory actions of A_2A_R (reviewed in [15]). In human neutrophils, A_2A_R activation depresses the expression of inflammatory mediators such as leukotriene B_4_ and inflammatory cytokines, particularly TNF [6, 7, 9], while potentiating the COX-2 dependent generation of prostaglandin E_2_ [16]. Study of the involvement of A_2A_R in normal aging has been confined largely to effects on nervous tissues [17], while the relationship between aging and the clear role for immune-cell-borne A_2A_R in the resolution of inflam-mation has received little attention [18].

In the present study, we examined the impact of aging on an acute inflammatory response. We measured neutrophil influx, viability, activation and gene expression as well as cytokine accumulation in dorsal air pouches raised on wild-type (WT) and A_2A_R-knock-out (KO) mice. Results reveal that the quality of several neutrophil-dependent local responses declines with age and that functional A_2A_R provides some protection against this decline.

## RESULTS

**Figure 1:**
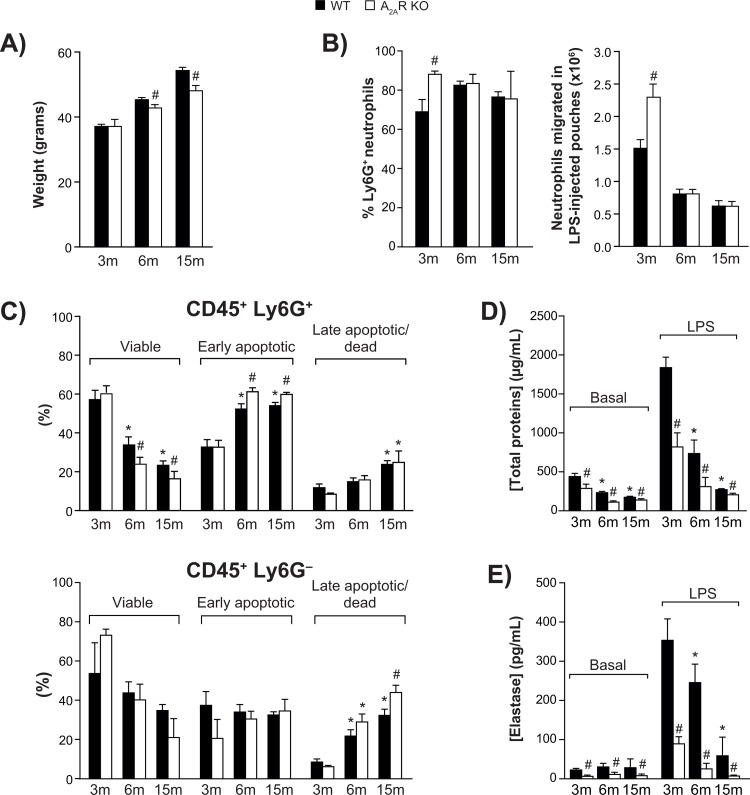
Animal body weight, air pouch leukocyte counts, and viability (**A**) Body weight of wild-type (WT) and A_2A_R-knockout mice aged 3, 6 and 15 months (*n* = 30 per group). (**B**) Ly6G+ neutrophils among cells recovered from LPS-injected dorsal air pouches (*n* = 12 mice per group) were enumerated as described in Methods. (**C**) Ly6G-positive (+) neutrophil (top panel) and Ly6G-negative (−) leukocytes (bottom panel) viability was assessed as described in *Methods*. (*n* = 12 mice per group). All values are expressed as mean ± SEM. *Significantly different from 3 months within a genotype. ^#^Significantly different from the age-matched WT group. (**D-E**) Cell-free exudates were analyzed for total protein and neutrophil elastase concentrations. Phosphate-buffered saline (Basal) or buffer + LPS was injected into air pouches raised on wild-type (WT) and A_2A_R-knockout mice (aged 3, 6 or 15 months) as described in *Methods*. All values are expressed as mean ± SEM for *n* = 12 mice per group. *Significantly different from 3 months within a genotype. ^#^ Significantly different from the age-matched WT group.

A shown in Figure 1A, the mice gained weight steadily over the experimental period. Body weight was significantly smaller in A_2A_R-KO mice at the age of 6 months, and these animals were 12 % lighter than the WT group at the age of 15 months.

### Aging has a negative impact on the accumulation and viability of neutrophils in the air pouch

Injection of LPS into a dorsal air pouch (500 ng/pouch) elicits the recruitment of neutrophils [8]. Fractions of Ly6G^+^ neutrophils remained relatively constant throughout age groups, ranging from 70% to 82% in WT mice (Fig. 1B, left panel). In 3-month-old WT mice, approximately 1.5 × 10^6^ neutrophils had accumulated 4 h after injection (Fig. 1B, right panel). Neutrophil infiltration was 52 % greater in KO mice. However, by the age of 15 months, the numbers had dropped by more than half for both genotypes. No such decrease was observed in the bloodstream of WT mice: counts of leukocytes, including lymphocytes, monocytes, and neutrophils, were all even higher at 15 months (Suppl. Fig. S1). The LPS injection caused bloodstream neutrophil counts to double in young and old mice, while lymphocyte and monocyte counts were unchanged. The viability of Ly6G^+^ neutrophils harvested from the air pouches dropped as the mice aged (Fig. 1C, top panel). This occurred faster in KO mice, in which the final counts were approximately 30 % lower than in WT, and is reflected in the proportions of early apoptotic neutrophils and late apoptotic/dead cells. Increased neutrophil apoptosis in A_2A_R-KO mice is consistent with earlier reports indicating that activation of A_2A_R delays apoptosis in human neutrophils [19, 20]. The viability of non-neutrophil leukocytes (Ly6G^−^) similarly decreased with aging (fig 1C, bottom panel).

### Local tissue permeability and neutrophil degranulation decrease with aging

The permeability of local interstitial tissues decreased markedly as the animals aged, based on the total protein content of the air pouch fluid, both in saline-injected (basal condition) and LPS-injected animals (Fig. 1D). In WT mice injected with LPS, total protein decreased by 83 % between the ages of 3 months and 15 months. The corresponding decrease in KO mice was more pronounced. Measured as an indication of neutrophil azurophilic degranulation [21], elastase activity also decreased as the mice aged (Fig. 1E), noticeably more in KO mice.

### Aging affects the profile of cytokine accumulation at the inflammatory site

**Figure 2:**
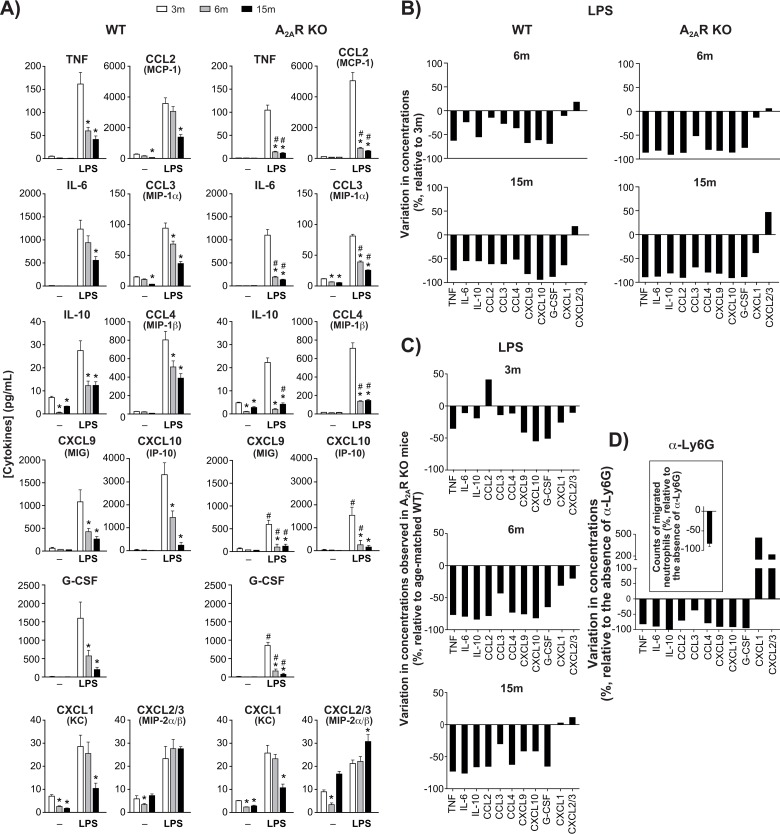
Cytokine/chemokine levels in dorsal air pouches (**A**) Concentrations of cytokine, chemokine or growth factor in cell-free exudate recovered from WT and A_2A_R KO mice after injecting air-pouches with phosphate buffered saline (−) or with buffer + LPS. Mice were aged 3, 6 or 15 months (m). Concentrations were measured using a multiplex immunoassay as described in Methods and are expressed as mean ± SEM for *n* = 12 mice. *Significantly different from the concentration measured at 3m. ^#^ Significantly different from the age-matched wild-type group. (**B**) Changes (%) in concentrations measured at 6m and 15m relative to 3m, in LPS-injected air pouches. (**C**) Variations between wild-type and A_2A_R-knockout mice concerning cytokine concentrations measured in LPS-injected dorsal air pouches. Values are % changes relative to age-matched WT. (**D**) The effect of neutrophils influx on cytokine/chemokine levels in dorsal air pouches. WT mice aged 3m were injected with anti (ɑ)-Ly6G before injection of LPS into air pouches. Cytokine concentrations and neutrophil counts (insert) in the pouches are expressed as the change (%) relative to control mice (not injected with the antibody). Values are expressed as mean ± SEM (n = 5).

Levels of each cytokine and chemokine measured in air pouch exudates of WT and KO mice at three months of age were for the most part comparable, whether the mice were injected with LPS or not (Fig. 2A). However, as the animals aged, levels of TNF, IL-6, IL-10, CXCL1, and CCL2-4 and G-CSF decreased markedly while CXCL2-3 remained elevated. Figure 2B illustrates the changes observed respectively in LPS-injected WT and KO mice, relative to cytokine status at 3 months. The decrease was faster and greater in KO mice than in WT mice, with levels of CXCL 1, 2 and 3 dropping more slowly. An age-matched comparison between the two genotypes shows that the effect of A_2A_R knockout was most apparent at the age of 6 months and that CXCL 1, 2 and 3 were the least affected (Fig. 2C). Additional cytokines and growth factors were measured, including IL-1ɑ, IL-1β, IL-2 to IL-5, IL-7, IL-9, IL-12, IL-13, IL-15, IL-17, LIF, M-CSF, GM-CSF, IFN-γ, and VEGF, but their con-centrations were stable and remained below 50 pg/ml consistently under all conditions tested (data not shown).

Such clear age-related decreases in cytokine concentrations were not observed in the bloodstream. Basal circulating levels remained quite constant over the 1-year period of study (*Suppl. Fig S2*). Only IL-15 and to a lesser extent CXCL 1, 2 and 3 and CCL 2 and 3 tended to decrease with age, but not to a significant extent. Injection of LPS into the pouch increased the levels of several cytokines in the bloodstream. The highest levels measured were by far for G-CSF, in young and older mice. Levels of IL-6, CXCL9 (MIG), CXCL10 (IP-10), CCL2 and to a lesser extent CCL4 also increased. For most analytes, blood levels in LPS-injected mice were comparable at 3 and 15 months. Exceptions included IL-6, which increased significantly in older mice, while levels of G-CSF and CXCL10 decreased. Levels of CCL3, CCL4, and IL-15 tended to increase in older mice but did not reach significant levels. IFN-γ, IL-2, IL-3, IL-4, IL-5, IL-7, IL-9, IL-10, IL-12, IL-13, IL-17, LIF, M-CSF, and VEGF were stable and consistently below 100 pg/ml under all tested conditions.

### Incoming neutrophils are required for normal cytokine production at the site of inflammation

To hinder neutrophil migration to the inflammation sites, 3-month-old WT mice that were to be treated with LPS received an intravenous injection of anti-Ly6G, which works via a β2-integrin-dependent mechanism [22]. Neutrophil counts in LPS-injected pouches were thus decreased by as much as 80 % (Fig. 2D, *insert*). Subsequent accumulation of TNF, IL-6, IL-10, CCL2-4, CXCL9-10 and G-CSF was also decreased, often by more than 75 % (Fig. 2D), while levels of CXCL 1, 2 and 3 increased by 2–4 fold. Preventing neutrophil migration thus appears to replicate the effects of aging on the local accumulation of cytokines.

### Aging affects gene expression profiles in migrated leukocytes

**Figure 3:**
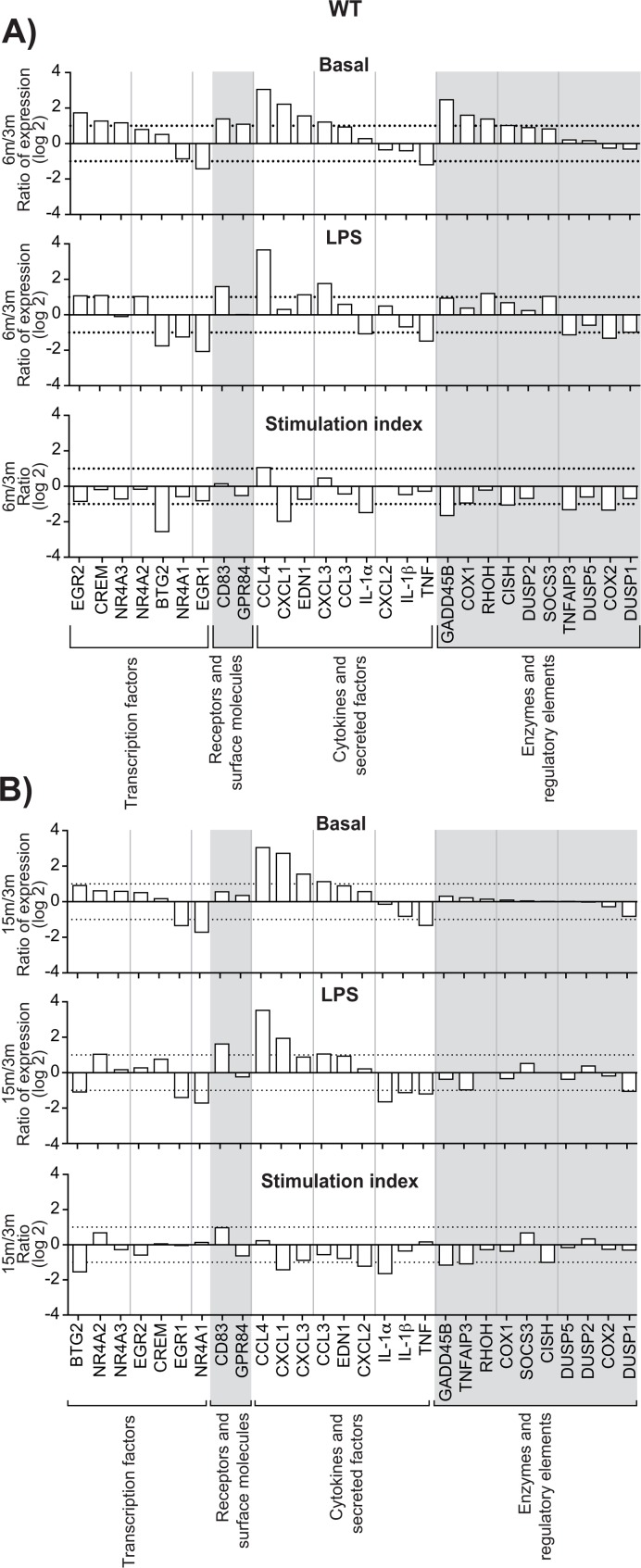
Effect of age on gene expression in leukocytes recovered from dorsal air pouches raised on WT mice Gene mRNA transcripts were quantitated as described in Methods and categorized as transcription factors, receptors, cytokines or enzymes. In each category, genes are ranked from the largest increase to the largest decrease under basal conditions (saline injection only). (**A**) 6‐month to 3‐month age comparison. (**B**) 15‐month to 3‐month comparison. “Stimulation index” is the change due to stimulation by LPS injection, relative to the basal condition. Dotted lines indicate one doubling or halving of gene expression. Values are expressed as the base‐2 logarithm.

**Figure 4:**
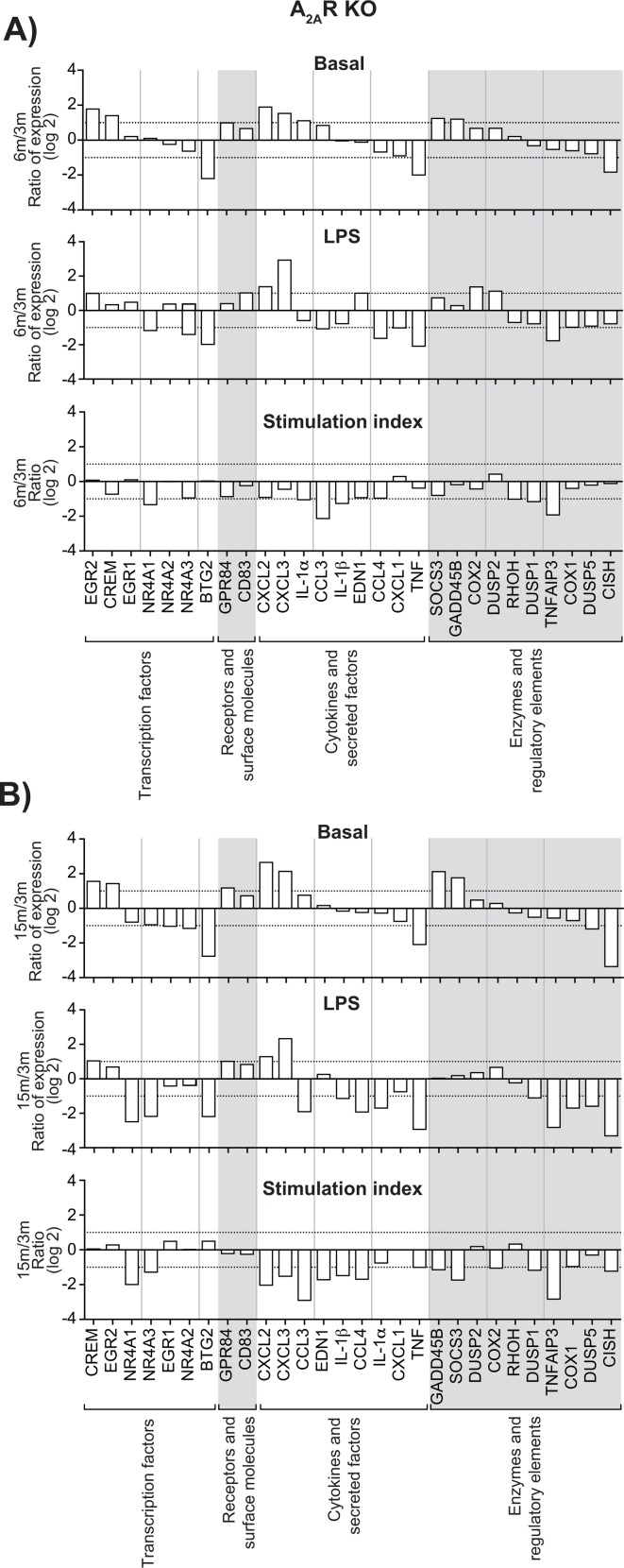
Effect of age on gene expression in leukocytes recovered from dorsal air pouches raised on A_2A_R KO mice Please refer to the legend for Figure 3

**Figure 5:**
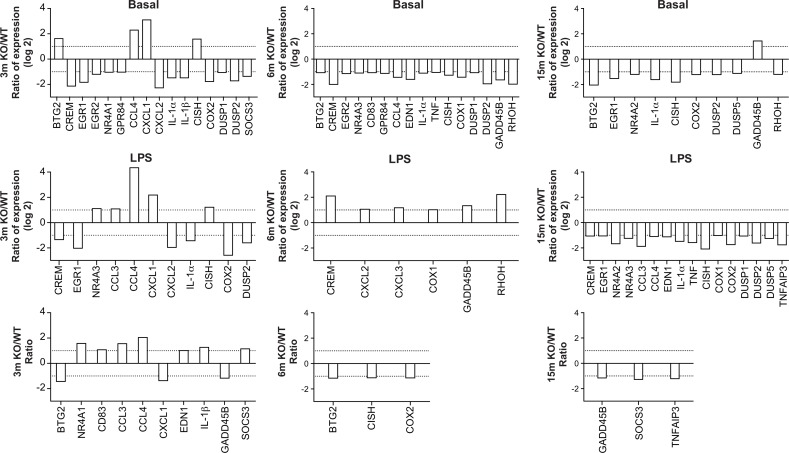
Age-matched comparisons of gene expression in leukocytes recovered from dorsal air pouches raised on wild-type (WT) and A_2A_R-knockout (KO) mice Ratios were determined for the basal condition (injection of PBS only, top row), the LPS-stimulated condition (middle) and the stimulation indexes (bottom).

Increased expression of genes encoding transcription factors, cytokines, enzymes, regulatory elements and receptors is an additional indication of leukocyte activation [7, 8]. We calculated the stimulation indexes of various genes in neutrophils as a function of age under basal and inflamed (LPS) conditions, WT and KO mice (Suppl. Figs. 3 & 4). Consistent with our previous observations [8], mRNA encoding the cytokines IL-1β, CXCL2, CCL 3 & 4 and phosphatase DUSP1 were among the most abundant, under basal and LPS-inflamed conditions. Messenger RNA encoding IL-1ɑ, IL-1β, CCL 3 & 4, CXCL 2 & 3, MAPK signaling factor GADD45B and suppressor signals SOCS3 and TNFAIP3 had the highest stimulation indexes. CCL3, CCL4, CXCL1, and CXCL3 mRNA levels increased in leukocytes recovered from air-pouches of WT mice aged 6 and 15 months, under basal and LPS conditions, by more than 10-fold in the case of CCL4 (Fig. 3A & B, top and middle panels). In contrast, TNF mRNA diminished consistently. IL-1ɑ mRNA faded under conditions of LPS stimulation. The CCL4 stimulation index increased at six months, while indexes for CXCL1 and IL-1ɑ decreased (Fig. 3A & B, bottom panels). Increases in CXCL2 and CXCL3 and decreases in TNF under basal and LPS conditions were greater in KO than in WT mice (Figure 4A & B). Stimulation indexes of IL-1ɑ, IL-1β, CCL3, CCL4, CXCL2, CXCL3, EDN1, and TNF were affected negatively as the animals aged. Age-matched com-parisons between KO and WT revealed CCL4 expression up to 16 times higher in leukocytes recover-ed from dorsal air pouches of KO compared to WT mice (Fig. 5), while CXCL2 and IL-ɑ decreased. Stimulation indexes for CCL3, CCL4 were higher. In 15-month-old mice, the vast majority of the affected genes were expressed more weakly in KO than in WT mice. A compilation of the most salient gene expression trends is presented in Table 1.

**Table 1:** Genes most affected by aging in leukocytes harvested from LPS-injected air pouches

	WT		A_2A_R-KO		
**Up-regulated**			**Up-regulated**		
At 6m, both in basal and LPS conditions	At 15m, both in basal and LPS conditions	Stimulation indexes (6m or 15m)	At 6m, both in basal and LPS conditions	At 15m, both in basal and LPS conditions	Stimulation indexes (6m or 15m)
**CCL4**	**CCL4**	CCL4	**CXCL2**	**CXCL2**	-*None*
**CXCL3**	**CXCL3**		**CXCL3**	**CXCL3**	
**EDN1**	**EDN1**		EGR2	CREM	
CREM	CCL3				
EGR2	CXCL1			GPR84	
CD83					
GADD45B					
**Down-regulated**			**Down-regulated**		
At 6m, both in basal and LPS conditions	At 15m, both in basal and LPS conditions	Stimulation indexes	At 6m, both in basal and LPS conditions	At 15m, both in basal and LPS conditions	Stimulation indexes
**EGR1**	**EGR1**	**BTG2**	**BTG2**	**BTG2**	**NR4A1**
**NR4A1**	**NR4A1**	**IL-1ɑ**	**TNF**	**TNF**	**NR4A3**
**TNF**	**TNF**	**CISH**	CXCL1	CISH	**CCL3**
		**GADD45B**		DUSP5	
		**TNFAIP3**			**CCL4**
		**CXCL1**			**CXCL2**
					**CXCL3**
					**EDN1**
					**IL1β**
					**DUSP1**
					**TNFAIP3**

Genes expressed differentially in 6-month-old and 15-month-old mice, based on comparison to genotype-matched 3-month-old mice. Criterion: gene expression was at least doubled (Up-regulated), or halved (Down-regulated), compared to the expression at 3m.

In **bold**: affected both at 6m and 15m.

## DISCUSSION

The air pouch model of inflammation is a powerful tool for *in vivo* studies of cell activation and migration of leukocytes to lesions [8]. It allows easy collection of exudates and analysis of mediators of inflammation. Cells of tissues lining the air pouch are activated and release TNF and chemokines of the CXC family, which attract neutrophils [23]. Among the most important factors in this process are CXCL 1, 2 and 3 [24]. Neutrophils accumulating in the lesion may then become a significant source of chemokines and cyto-kines that contribute to a proper inflammatory response and its subsequent resolution [23].

A common age-associated chronic increase in inflammatory markers appears in some cases to bring about exaggerated inflammatory responses [25-27]. However, in the present model of sub-dermal injury, we showed an age-related decline in salient aspects of the local inflammatory response, including edema and leukocyte accumulation, viability and activation in the dorsal air pouch. This was not due to lower numbers of leukocytes in circulation. Aging also affected the profile of cytokines, chemokines and growth factors accumu-lating at the site of lesion, in particular, TNF, IL-6, CCL3, CCL4, and G-CSF, while levels of neutrophil-attracting CXCL2/3 were unaffected. Our results obtained in mice are consistent with observations of age-related fading of neutrophil function, made in human subjects [10, 28]. These include decreases in extracellular trap formation, phagocytosis, degranula-tion, ROS production and microbicidal capability [3], all of which weaken host defenses. Also, neutrophil chemotaxis is correlated with longevity in asymp-tomatic aged individuals [29]. As well, the increased susceptibility of aged subjects to infections appears not to be due to an exaggerated inflammatory response, but rather to an ineffective innate immune response [30]. Neutrophils from elderly patients have been found to respond poorly following TREM-1 engagement, suggesting possibly an important intrinsic cause of the higher incidence of sepsis-related deaths in this sector of the population [31]. Such neutrophils also exhibit a reduced respiratory burst priming and activation capacity in response to granulocyte-macrophage colony-stimulating factor (GM-CSF) and a reduced capacity to delay apoptotic events [32]. Mechanisms that mediate these age-related defects have yet to be identified. A study conducted in aging rats reported that neutrophil themselves remain responsive, while a plasma protein interacting with neutrophil receptors for complement-derived chemoattractants might influence neutrophil responses to infection and inflammation in the elderly [33]. More recently, Hazeldine et al. observed an impaired LPS-induced neutrophil extra-cellular trap formation in aged individuals while TLR4 expression was not affected, suggesting a defect in proximal signaling to explain the age-related decline [11]. Although genetic and environmental influences and the complexity of the immune system are likely involved, the mechanisms that cause age-associated imbalances remain unclear. Moreover, our results around neutrophil decaying responses must be taken in a larger context of age-related immune and host defense decline. Insufficient innate responses in aging may lead to longer, or even chronic, inflammatory episodes causing harm to tissues [34, 35] and promoting inflam-mation further. Nonetheless, an ineffective inflammato-ry response is clearly at play in older subjects.

A key observation in the present study is that preventing neutrophil migration to the lesion by immunological means largely reproduced in young mice the effects of aging on local cytokine/chemokine accumulation, implying that neutrophils are the principal source of cytokines found in the air pouch. However, support for such a role is scant, and it has been shown that cytokines such as TNF can accumulate before neutrophil infiltration [23]. What our results do indicate is that unhindered accumulation of neutrophils promotes the optimal production of local mediators of inflammation [23] and that deficient migration to the lesion might be a significant cause of the diminished responses seen in aging individuals. The cytokine G-CSF, a factor involved directly in neutrophil formation, mobilization and activation [36], was one of the few analytes for which blood concentrations diminished significantly as the mice aged. Whether or not this explains the sluggish migration of neutrophils in older animals remains to be demonstrated. It nonetheless raises the possibility that imbalances extrinsic to neutrophils contribute to altering cellular responses as animals age. A better understanding of the roles of such factors will likely be important in the treatment of age-related immune dysfunction.

Aging also affected the gene expression profile of leukocytes recovered from the air pouch. Messenger RNA encoding the monocyte-chemoattractants CCL4 (MIP-1β) and to a lesser extent CCL3 (MIP-1ɑ), al-ready ranking among the most abundant transcripts in resting or stimulated polymorphonuclear cells, increased more in aging WT mice. One of the critical chemokines for granulocyte recruitment, namely CXCL3, increased in both genotypes, suggesting an attempt to compensate for otherwise insufficient leukocyte migration. These results suggest that under conditions of unhindered migration, a yet unidentified feedback signal is generated to modulate the transcription of genes encoding leukocyte-attracting chemokines. In both genotypes, TNF mRNA was among the transcripts that decreased most in abundance with aging, which is consistent with a diminished inflammatory response, given the important role of this cytokine.

To our knowledge, the present study is one of the first to address the effects of aging on A_2A_R-dependent neutrophil responses [18]. The results show that the effects of aging are more pronounced in A_2A_R-KO mice. At all ages examined, neutrophil viability was decreased in mice devoid of functional A_2A_R, confirming previous findings of an anti-apoptotic role for A_2A_R in human neutrophils [19, 20]. Moreover, total proteins, neutrophil elastase, and cytokines were all decreased significantly in air pouches in older A_2A_R-KO mice, which is consistent with increased neutrophil death. While many readouts were affected in A_2A_R-KO mice, differences between age-matched genotypes were more subtle than those caused by aging. While it is too early to speculate on the physiological consequences of A_2A_R-deficiency in aging, the trend in worsening effects of aging remains intriguing. A_2A_R is recognized as a termination signal in several *in vitro* and *in vivo* models of the acute inflammatory response [14, 15, 37]. In young mice without a functional A_2A_R, pro-inflammatory signals reach higher concentrations or persist for longer periods of time [14, 38]. This has been shown for local production of TNF in 6–8 week-old mice [6]. The absence of such a pivotal stop-signal pathway is likely to cause prolonging of inflammatory episodes, which might eventually desensitize portions of the immune system. This is consistent with the concept of inflammaging, in which chronically elevated concentrations of inflammatory markers lead to the diminishing of neutrophil-associated inflammatory responses [39]. Proper termination of local inflam-matory responses by functional A_2A_R might thus contribute to maintaining immune response efficiency as the subject ages. A_2A_R deficiency also affected bodyweight in aging, presuming effects beyond neutrophil responses. Studies focused on low-level chronic inflammation as a possible contributing factor to diminishing acute inflammatory response in aging subjects are in progress in our laboratory.

In conclusion, aging brought a clear decrease in some important aspects of a local acute inflammatory response in mice, including neutrophil migration, viability, and activation. Sluggish neutrophil migration itself appeared to be a possible causative agent. The absence of the A_2A_R stop-signaling pathway worsened much of the observed decline associated with aging, indicating the importance of a proper resolution process for maintaining the effectiveness of innate immune responses as age advances.

## MATERIALS AND METHODS

### Experimental design

A_2A_R heterozygotes (A_2A_R^+/−^) CD1 mice were paired. Offsprings were genotyped to select A_2A_R^−/−^ (KO) and A_2A_R^+/+^ (wild-type) animals, as described previously [6]. Mice were kept for up to 15 months in groups of 4 per cage at 20°C and 60 % relative humidity with a light-dark cycle of 12 h. Access to food and water was *ad libitum*. Age groups (3, 6, 15 months old), genotypes and age-matched genotypes were compared. The dependent variables were: counts and viability of granulocytes recovered from the dorsal air pouch, expression (mRNA) of inflammatory genes in leuko-cytes and in the tissue lining the air pouch, accumulation of cytokines/chemokines and elastase activity in the pouch exudates.

### Dorsal air pouches

The Université Laval animal protection committee approved all air-pouch experiments. These were conducted as described previously [8]. Briefly, dorsal pouches (one per mouse) were raised by subcutaneous injection of 5 mL of sterile air on day 0 and 3 mL on day 3. On day 6, individual air pouches were injected with 500 μL of pre-warmed, endotoxin-free phosphate-buffered saline (PBS), or PBS containing 500 ng of lipopolysaccharide from *Escherichia coli* (LPS, *E. coli* 0111:B4, Sigma-Aldrich Canada Co., Oakville, ON, Canada). Mice were sacrificed 4 h after LPS injection, and air pouches were washed twice with ice-cold PBS (total of 2 mL). Leukocyte suspensions were assayed for cell enumeration with a Cellometer Auto T4 Plus cell counter (Nexcelom Bioscience LLC, Lawrence, MA, USA). Blood was collected by cardiac puncture. The experimental groups contained an average of 8 mice and equal numbers of females and males. The entire experiment was repeated five times.

### Cell subtyping

Identification of leukocyte and subtypes were performed using V450-conjugated rat anti-mouse CD45 IgG2b, k (leukocyte marker), APC-conjugated rat anti-mouse Ly6G (1A8) IgG2a, k (granulocyte marker), FITC-conjugated rat anti-mouse CD3 IgG2b, k (BD Biosciences, Mississauga, ON, Canada; lymphocyte marker), and PE-Cyanine7-conjugated rat anti-mouse CD115 IgG2a, k (eBioscience, San Diego, CA, USA; monocyte marker). Briefly, 100 μL of cell suspension was incubated with 0.2 μg of anti-CD45, 0.2 μg of anti-Ly6G, 0.5 μg of anti-CD3 and 0.2 μg of anti-CD115 for 30 min in the dark. PBS was added (400 μL) and samples were analyzed using a FACS Canto II flow cytometer with FACSDiva software, version 6.1.3 (BD Biosciences).

### Neutrophil viability

Cell viability was assessed using V450-conjugated rat anti-mouse CD45, APC-conjugated anti-Ly6G, and a FITC Annexin V Apoptosis Detection Kit (BD Biosciences). Briefly, 100 μL of cell suspension was incubated with 0.2 μg each of anti-CD45 (leukocyte marker) and anti-Ly6G for 30 min in the dark. After centrifugation, cell pellets were suspended in 100 μL of the binding buffer 1X provided with the apoptosis detection kit. Annexin V (5 μL) and 5 μL of propidium iodide were added to each sample. After 15 min, 400 μL of binding buffer was added, and samples were analyzed using a FACS Canto II flow cytometer with FACSDiva software, version 6.1.3 (BD Biosciences). Gating was determined using control samples labeled individually with either Annexin V or propidium iodide.

### Elastase, and total proteins

Mouse elastase and total proteins in air pouch exudates were measured using respectively an ELISA method (R&D Systems, Minneapolis, MN, USA) and a protein assay (Bio-Rad, Mississauga, ON, Canada) per the manufacturers' instructions.

### Metabolites

Cytokine/chemokine levels in cell-free supernatants recovered from dorsal pouch exudates were measured using a multiplexed bead-based immunoassay (BD™ Cytometric Bead Array) according to the manufacturer's protocol. TNF (C8), IL-6 (B4), IL-10 (C4), IL-1β (E5), GM-CSF (B9), CCL2/MCP-1 (B7), CCL3/MIP-1ɑ (C7), CCL4/MIP-1β (C9) and CXCL1/KC (A9) levels were determined using a FACS Canto II flow cytometer with FCAP Array software, version 3.0 (BD Biosciences). CXCL2-3/MIP-2 measurements were per-formed using a commercially available ELISA kit (R&D systems Inc., Minneapolis, MN, USA) according to the manufacturer's instructions. Plasma cytokines and G-CSF were measured using Eve Technologies (Calgary, AB, Canada).

### RNA isolation

Total RNA was isolated from approximately 5×10^6^ leukocytes (pellet of centrifuged dorsal pouch cell suspension) using Ribozol™ (Amresco, Solon, OH, USA) according to the manufacturer's protocol, with modifications [8, 9]. Briefly, the sample was homogenized in 1 mL of Ribozol™, and 200 μL of chloroform were added. Samples were mixed and then centrifuged at 12,000 x *g* for 15 min (4°C), and the aqueous phase (450 μL, on top) was transferred to a tube containing an equal volume of isopropanol, mixed thoroughly using a vortex device and centrifuged at 12,000 x *g* for 10 min (4°C). The supernatant was discarded, and the precipitated RNA pellet was washed twice in 500 μL of 75 % ethanol with centrifugation at 12,000 x *g* for 5 min (4°C). The final pellet was allowed to air-dry for 5-10 min and then re-suspended in RNase-free water. RNA was quantitated using a Qubit^®^ Fluorometer (Life Technologies Inc., Burlington, ON, Canada).

### Comparative real-time PCR

Gene expression (mRNA transcript abundance) was monitored in leukocytes using real-time PCR. Reverse transcription was performed using 1 μg of total RNA with a Transcriptor First Strand cDNA Synthesis Kit (Roche Applied Science, Laval, QC, Canada) following the manufacturer's instructions. Real-time PCR was performed as described previously [40]. Briefly, cDNA amplification was carried out in a Rotor-Gene Q operated with Q-series software version 2.0.2 (Qiagen Inc, Mississauga, ON, Canada) using 35 cycles of 95°C for 17 seconds, 58°C for 25 seconds and 72°C for 25 seconds. Each reaction mixture contained 40 ng of cDNA, 2 μL of 10X buffer (100 mM Tris, 500 mM KCl, 30 mM MgCl_2_, 1.5 % Triton X-100), 100 μM dNTP, 500 nM of primers, 0.1 unit of *Taq* DNA polymerase (Roche Applied Science) and SYBR^®^ Green I dye (Life Technologies Inc.) diluted 1:30 000 in a volume of 20 μL. Reaction specificity was ascertained by performing the Melt^®^ procedure (58–99°C, 1°C per 5 s) at the end of the amplification protocol, according to the manufacturer's instructions. For each gene of interest, specific primers were designed as described previously [8]. Briefly, primers were selected systematically within the coding region, with a theoretical melting point of 58°C, GC content of 50 % (± 10 %) and 18–24 base pair length, for an average product length of 200 base pairs. Primers thus designed were all tested with gradient PCR before use in real-time PCR [8]. Internal control genes were ranked using RefFinder, a web-based tool (http://150.216.56.64/referencegene.php?type=reference) developed for reference gene screening and evaluation based on published datasets and four ranking methods [8]. The RefFinder overall final ranking of each gene is a weighting calculated as the geometric mean of the rankings obtained from each method. Six genes were selected as candidate control genes: GAPDH, GUSB, H2AFZ, PPIA, TUBB4A, and UBC. The gene with the best ranking was used as the normalizing factor for presenting relative mRNA expression. In this study, GAPDH consistently ranked best.

### Statistical analysis

Where applicable, values are expressed as mean ± SEM. Unless stated otherwise, statistical analyses were performed using Student's two-tailed unpaired *t*-test. The criterion for declaring a difference to be significant was *p* < 0.05.

## SUPPLEMENTARY MATERIAL FIGURES


