# Supplementary material for: Age-related decline of the acute local inflammation response: a mitigating role for the adenosine A2A receptor
Source: Aging (Albany NY). 2017 Oct 18;9(10):2083–97. doi: 10.18632/aging.101303 (PMC5680557; doi:10.18632/aging.101303)
Supplement: Supplementary file 1 [file aging-09-2083-s001.pdf]

SUPPLEMENTARY MATERIAL

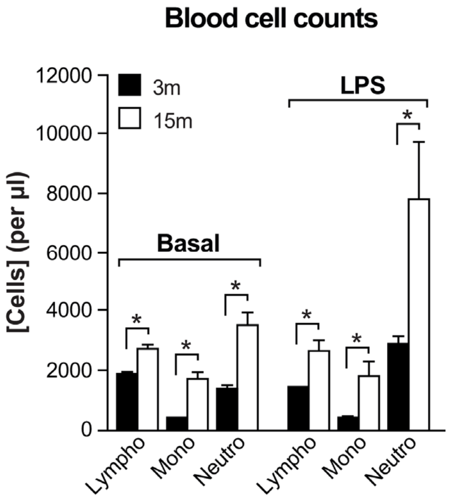

Supplementary Figure S1. Blood cell counts. Dorsal air pouches raised on wild-type (WT) mice aged 3 or 15 months were injected with either saline (Basal) or LPS for 4 h. Circulating lymphocytes, monocytes, and neutrophils were then enumerated as described in Methods. Results are expressed as mean ± SEM for *n* = 12 mice per group. *Significantly different.

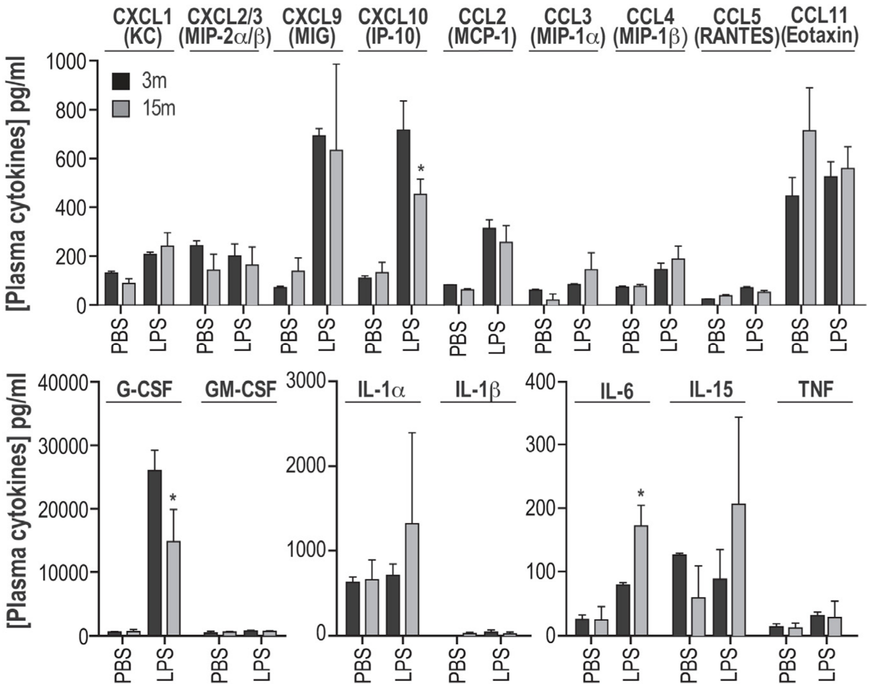

Supplementary Figure S2. Plasma cytokine levels. Dorsal air pouches raised on wild-type (WT) mice aged 3 or 15 months were injected with either saline (Basal) or LPS. Circulating plasma cytokine levels reached 4 h later were measured as described in Methods. Results are expressed as mean ± SEM for *n* = 12 mice per group. *Significantly different.

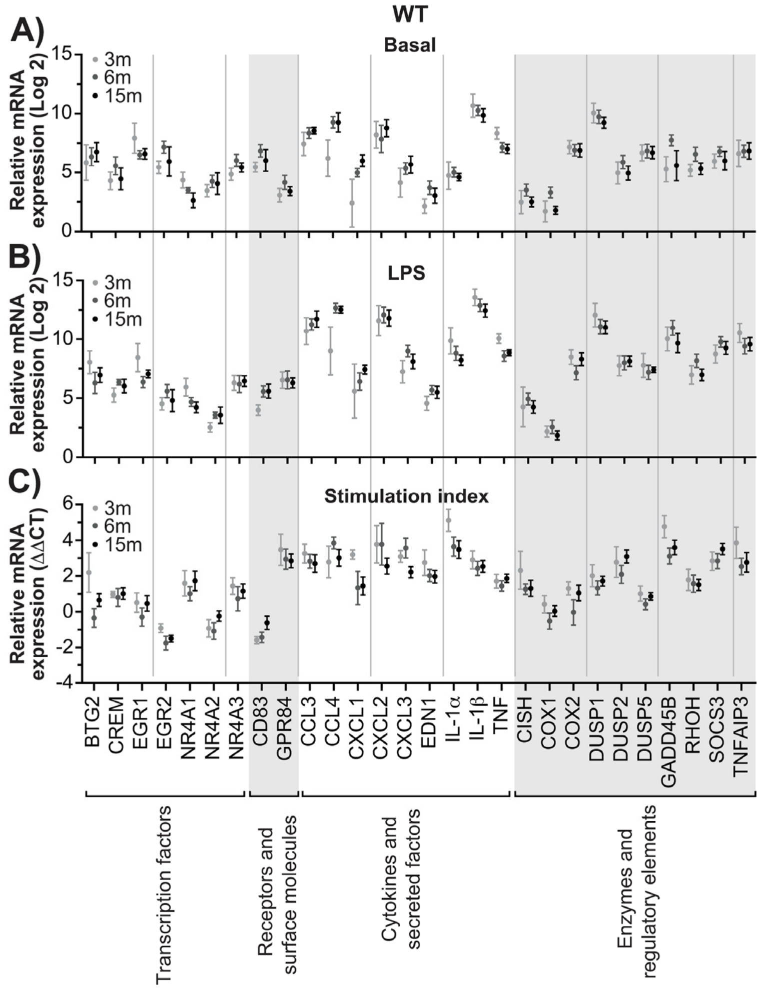

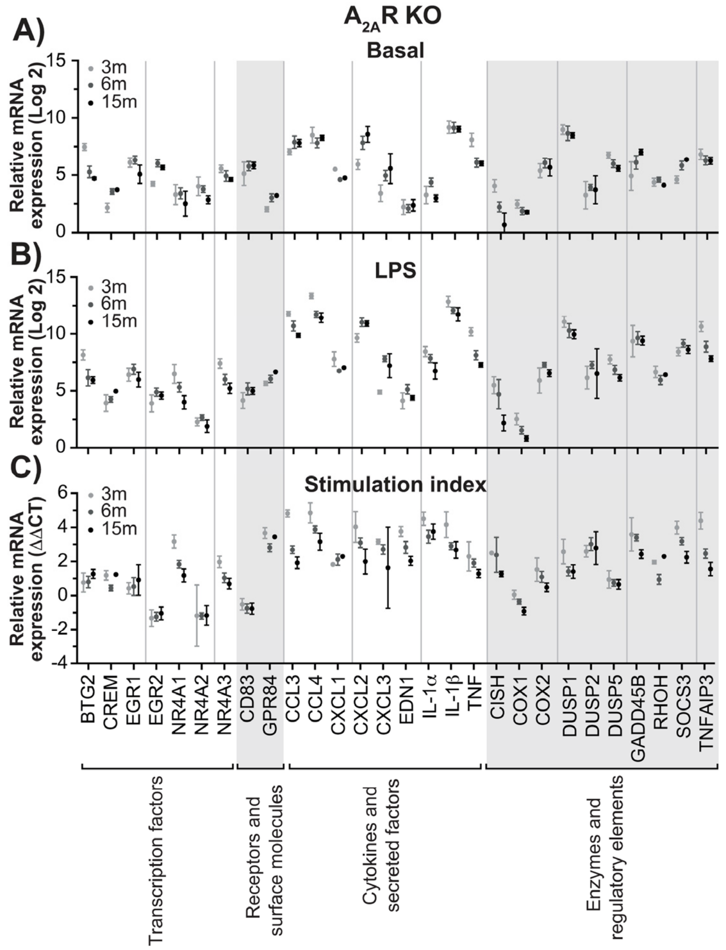

Supplementary Figure S3. Gene expression in leukocytes recovered from dorsal air pouches raised on wild-type (WT) mice. The abundance of mRNA transcript 4h after injection with saline (Basal) or LPS was measured for each gene as described in *Methods*. (**A**) Basal; (**B**) LPS; (**C**) Ratios of stimulated to basal expression levels (stimulation index). Values were calculated from the mean threshold cycle ± SEM (cells from 8 mice were pooled for each value, *n* = 5 experiments), relative to COX-1 in 3-month-old WT mice.

Supplementary Figure S4. Gene expression in leukocytes recovered from dorsal air pouches raised on A_{2A}R-knockout (KO) mice. Please refer to the legend for Suppl. Fig. S3.
